# Nasogastric or nasojejunal feeding in predicted severe acute pancreatitis: a meta-analysis

**DOI:** 10.1186/cc12790

**Published:** 2013-06-20

**Authors:** Yu-sui Chang, Hua-qun Fu, Yuan-mei Xiao, Ji-chun Liu

**Affiliations:** 1 Department of Surgery, the First Affiliated Hospital of Nanchang University, No. 17, Yongwaizheng Street, Donghu District, Nanchang 330006, China; 2 Department of Surgery, the Second Affiliated Hospital of Nanchang University, No. 1, Mingde Road, Donghu District, Nanchang 330006, China; 3 Department of Occupational Health School of Public Health, Nanchang University, No. 463, Bayi Road, Donghu District, Nanchang 330006, China

**Keywords:** meta-analysis, severe acute pancreatitis, nutritional support, enteral nutrition, mortality, tolerance

## Abstract

**Introduction:**

Enteral feeding can be given either through the nasogastric or the nasojejunal route. Studies have shown that nasojejunal tube placement is cumbersome and that nasogastric feeding is an effective means of providing enteral nutrition. However, the concern that nasogastric feeding increases the chance of aspiration pneumonitis and exacerbates acute pancreatitis by stimulating pancreatic secretion has prevented it being established as a standard of care. We aimed to evaluate the differences in safety and tolerance between nasogastric and nasojejunal feeding by assessing the impact of the two approaches on the incidence of mortality, tracheal aspiration, diarrhea, exacerbation of pain, and meeting the energy balance in patients with severe acute pancreatitis.

**Method:**

We searched the electronic databases of the Cochrane Central Register of Controlled Trials, PubMed, and EMBASE. We included prospective randomized controlled trials comparing nasogastric and nasojejunal feeding in patients with predicted severe acute pancreatitis. Two reviewers assessed the quality of each study and collected data independently. Disagreements were resolved by discussion among the two reviewers and any of the other authors of the paper. We performed a meta-analysis and reported summary estimates of outcomes as Risk Ratio (RR) with 95% confidence intervals (CIs).

**Results:**

We included three randomized controlled trials involving a total of 157 patients. The demographics of the patients in the nasogastric and nasojejunal feeding groups were comparable. There were no significant differences in the incidence of mortality (RR = 0.69, 95% CI: 0.37 to 1.29, *P *= 0.25); tracheal aspiration (RR = 0.46, 95% CI: 0.14 to 1.53, *P *= 0.20); diarrhea (RR = 1.43, 95% CI: 0.59 to 3.45, *P *= 0.43); exacerbation of pain (RR = 0.94, 95% CI: 0.32 to 2.70, *P *= 0.90); and meeting energy balance (RR = 1.00, 95% CI: 0.92 to 1.09, *P *= 0.97) between the two groups. Nasogastric feeding was not inferior to nasojejunal feeding.

**Conclusions:**

Nasogastric feeding is safe and well tolerated compared with nasojejunal feeding. Study limitations included a small total sample size among others. More high-quality large-scale randomized controlled trials are needed to validate the use of nasogastric feeding instead of nasojejunal feeding.

## Introduction

Severe acute pancreatitis (SAP) is characterized by high mortality rates and is a potentially lethal disease requiring nutritional support [1]. Nutritional support is considered a key issue in the management of the hypercatabolism secondary to extended pancreatic and extrapancreatic inflammation.

Parenteral nutrition (PN), which has been associated with a greater complication rate [2], was the preferred route in the past. PN results in a major breakdown of the gut mucosal defense barrier with subsequent bacterial/endotoxin translocation, leading to sepsis and infections locally and at distant sites. Clinical prospective studies have shown that increased intestinal permeability correlates with increased levels of endotoxin and also with the grade of severity of pancreatitis [3,4].

Recently, convincing evidence has demonstrated that compared with PN, enteral nutrition (EN) significantly reduces infectious complications and mortality [5,6], results in decreased organ failure and surgical intervention rate [2] and provides significantly better glycemic control [7] in predicted SAP. EN may improve outcomes in these patients if given early [8]. Nutritional support using EN should be the preferred method in patients with SAP [2,9,10] as recommended by current guidelines [11].

EN can be given through either the nasogastric (NG) or the nasojejunal (NJ) route. While most studies have shown that NJ feeding is an effective method of providing EN for patients with SAP, there are also successful studies using NG feeding [12-14].

NJ tube placement is cumbersome because, although both fluoroscopy and endoscopy are highly effective for placement of small bowel feeding tubes, it can take an experienced operator up to 30 minutes to achieve post-pyloric placement of a small bowel feeding tube [15]. In contrast, NG tube placement is an easy bedside procedure. Therefore, NJ tube placement is expensive and inconvenient compared with NG tube placement.

Traditionally, it was believed that stimulation of pancreatic secretion by EN is detrimental. NG or duodenal feeding has been believed to increase the chances of aspiration pneumonitis [16] and stimulate pancreatic secretion [17] resulting in inefficient restoration of gut mucosal integrity, whereas NJ feeding did not.

The aim of nutritional support is to meet the patient's elevated metabolic demands as much as possible without stimulating pancreatic secretion while maintaining gut integrity [8]. The first meta-analysis and systemic review by Petrov *et al*. [18] was encouraging by showing no significant differences between NG and NJ feeding regarding safety and tolerance. This study was expected to contribute greatly to the establishment of an ideal nutrient feeding approach in patients with SAP. However, well-designed and sufficiently powered randomized controlled trials (RCTs) on NG versus nasointestinal feeding are required before early NG feeding can be established as a standard of care [19]. It is believed that small-scale clinical trials can lead to erroneous conclusions [20,21].

In order to compare tolerance and clinical outcomes between NG and NJ feeding, we performed a meta-analysis because adequately powered data derived from RCTs comparing NG and NJ feeding in SAP are scarce [12] and the ideal route for EN remains to be established. We used mortality, tracheal aspiration, diarrhea and exacerbation of pain as the primary outcomes to assess the impact of NG and NJ feeding in patients with predicted SAP because tracheal aspiration, diarrhea and exacerbation of pain are concerns in NG feeding and mortality is an important variable used in evaluating (a blank space should be deleted here) intervention effects in therapy.

## Materials and methods

### Search strategy

We used a multi-method iterative approach to identify relevant studies and we conducted a computerized literature search of the PubMed database from 1966 to October 2012 using the following search terms: severe acute pancreatitis AND nasogastric or nasojejunal AND nutrition or feeding. We also searched the Cochrane Central Register of Controlled Trials and the EMBASE (1980 to 2012) databases with the same terms. References that included information on EN were screened in an attempt to find other relevant articles. There were no restrictions on publication language.

### Study selection and data extraction

We defined the publications included in this meta-analysis using the following selection criteria: 1) study design: RCTs; 2) population: hospitalized patients with predicted SAP; and 3) intervention: NG versus NJ feeding. We used the following outcome variables: the primary outcome was mortality and at least one of the following variables: incidence of tracheal aspiration, diarrhea and exacerbation of pain; the secondary outcome was achievement of energy balance. A structured data abstraction form was used to ensure completeness and consistency of appraisal for each study. We extracted study characteristics, methodological variables, intervention, participant characteristics, clinical variables and outcome measures. Article selection and data extraction were conducted independently by two authors. All disagreements were resolved by discussion among these two authors and any of the other authors of the paper.

### Quality assessment and statistical analysis

The quality of the included trials was assessed using a Jadad score [22]. Meta-analysis was performed using the Cochrane Collaboration's Review Manager Software 5 (RevMan 5.0). The risk ratio outcomes are presented with 95% confidence intervals. Heterogeneity between trials was tested using the chi-square test, with *P *<0.10 indicating significant heterogeneity (difference) [23]. A random effects model and a fixed effects model were used in the presence and absence of statistical heterogeneity, respectively. We used a funnel plot to uncover potential publication bias.

## Results

**Figure 1:**
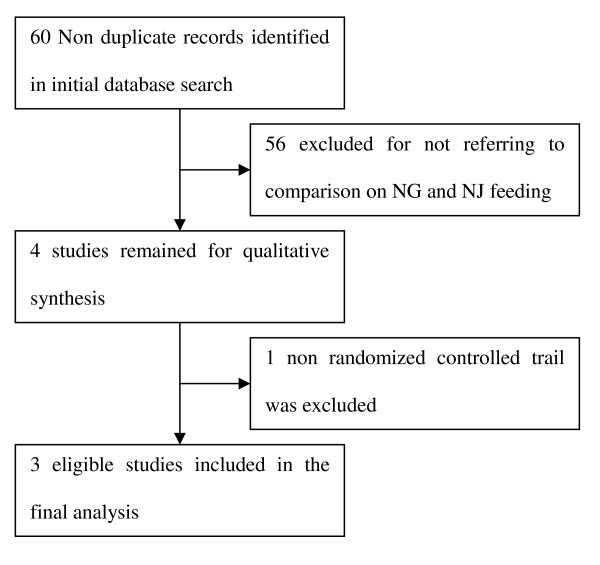
**Search, inclusion, and exclusion flow diagram**.

Sixty articles met the search criteria and 56 were excluded after screening to include only the studies comparing NG versus NJ or nasointestinal feeding. Of the four remaining articles, three were RCTs and one was a non-randomized cohort study [19]; therefore, three eligible RCTs were included in the analysis (Figure 1) [12-14]. The characteristics of the included studies are summarized in Table 1 and the quality of the included RCTs is shown in Table 2.

**Table 1:** Characteristics of included RCTs

Reference	Country	Design	Feeding start	Feeding formula	ITT method	Allocation concealment
Eatock 2005	UK	RCT	< 72 hours after onset	Semielemental	Yes	Adequate
Kumar 2006	India	RCT	48 to 72 hours of admission	Semielemental	Unclear	Unclear
Singh 2012	India	RCT	48 hours of admission	Semielemental	Yes	Adequate

ITT, intention-to-treat analysis; RCT, randomized controlled trial.

**Table 2:** Quality of included RCTs

Reference	Randomization method	Blind method	Withdrawal/drop-out	Jadad score
Eatock 2005	Computer generate random numbers	Not used	One excluded in NJ for misdiagnosed and two in NJ received NG for failure of NJ tube palcement	3
Kumar 2006	Computer generate random numbers	Not used	One excluded in NJ for failure of NJ tube placement	3
Singh 2012	Statistician generate random numbers	Not used	Two excluded in NJ for refusal of NJ tube re-insertion	3

The methodological quality of the randomized studies included was estimated using the criteria proposed by Jadad *et al*. [22]. NG, nasogastric; NJ, nasojejunal.

In the study by Eatock *et al*. [12], predicted SAP was defined as both the clinical and biochemical signs of acute pancreatitis and objective evidence of disease severity (Glasgow prognostic score of three or more or an Acute Physiology and Chronic Health Evaluation (APACHE) II score of six or higher or a C-reactive protein level greater than 150 mg/L). In the other two studies [13,14], predicted SAP was defined as a diagnosis of acute pancreatitis and at least one of three additional criteria: single or multiple organ failure as defined by the Atlanta classification; an APACHE II score of ≥8; and computed tomography severity index of ≥7. Overall, 157 patients with predicted SAP were enrolled in the included studies. Of these, 82 were randomly assigned to an NG group and 75 to an NJ group. Baseline demographic parameters of all subjects in the RCTs are shown in Table 3. There was no significant difference between the NG and NJ feeding groups at admission with respect to the demographic parameters, including multiple organ failure (MOF) and infected pancreatic necrosis (IPN). The baseline demographic data of the patients receiving the NG and NJ approaches were comparable. The main clinical outcomes of the NG and NJ groups in the included studies are shown in Tables 4 and 5, respectively. The severity of the patients at admission was comparable in the three included studies on the basis of the APACHE II score. The length of hospital stay (LOS) and duration of EN in the NG and NJ groups in all of the included studies were comparable.

**Table 3:** Baseline demographic parameters of total subjects in the RCTs

Parameters	Nasogastric feeding (number = 82)	Nasojejunal feeding (number = 75)	*P*
Gender			
Male	56	48	0.57
Female	26	27	
Etiology			
Biliary	36	42	
Alcohol	22	20	0.10
Idiopathic	16	12	
Others	8	1	
MOF	14	18	0.28
IPN	16	23	0.11
Mortality	14	18	0.25

IPN, infected pancreatic necrosis; MOF, multiple organ failure; RCTs, randomized controlled trials.

**Table 4:** Outcomes of patients receiving nasogastric feeding in the studies included

Study	Number of patients	Age (years)	APACHE II score	LOS (days)	Duration of EN (days)
Eatock 2005	27	63 (47 to 74)^a^	10 (7 to 18)^a^	16 (10 to 22)^a^	5
Kumar 2006	16	43.3 ± 12.8^b^	10.5 ± 3.8^b^	24.1 ± 14.4^b^	7
Singh 2012	39	39.1 ± 16.7^b^	8.5 (2 to 19)^a^	17 (1 to 73)^a^	7 or longer

Total	82	-	-	-	-

^a^Values are median (range); ^b^Values are mean±standard deviation; APACHE, Acute

Physiology and Chronic Health Evaluation; LOS, length of hospital stay.

**Table 5:** Outcomes of nasojejunally-fed patients in the studies included

Study	Number of patients	Age (years)	APACHE II score	LOS (days)	Duration of EN (days)
Eatock 2005	22	58 (48 to 64)^a^	12 (8 to 14)^a^	15 (10 to 24)^a^	5
Kumar 2006	14	35.6 ± 12.5^b^	9.6 ± 5.0^b^	29.9 ± 25.5^b^	7
Singh 2012	39	39.7 ± 12.3^b^	8 (2 to 24)^a^	18 (4 to 54)^a^	7 or longer

Total	75	-	-	-	-

^a^Values are median (range); ^b^Values are mean±standard deviation; APACHE, Acute

Physiology and Chronic Health Evaluation; EN, enteral nutrition; LOS, length of hospital stay.

**Figure 2:**
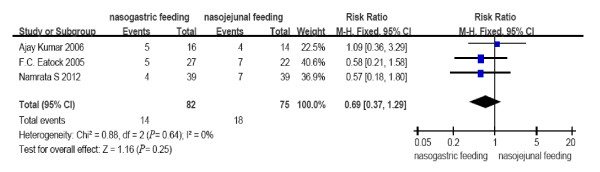
**Comparison of overall mortality between nasogastric feeding and nasojejunal feeding groups**.

**Figure 3:**
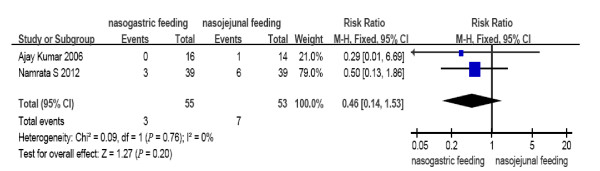
**Comparison of tracheal aspiration between nasogastric feeding and nasojejunal feeding groups**.

**Figure 4:**
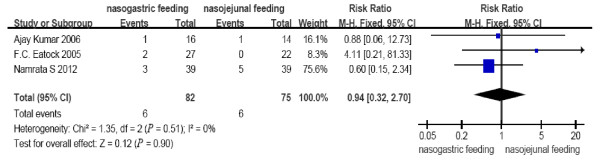
**Comparison of exacerbation of pain between nasogastric feeding and nasojejunal feeding groups**.

**Figure 5:**
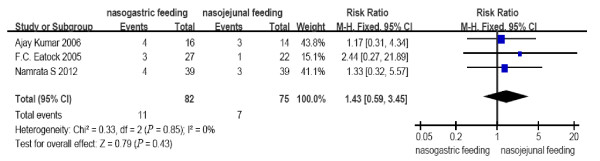
**Comparison of diarrhea between nasogastric feeding and nasojejunal feeding groups**.

**Figure 6:**
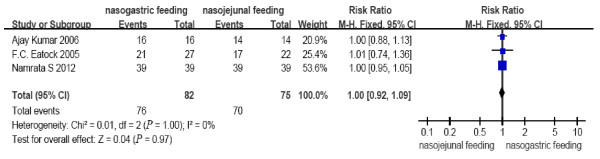
**Comparison of achievement of energy balance between nasogastric feeding and nasojejunal feeding groups**.

All the included RCTs reported the mortality, occurrence of diarrhea, exacerbation of pain and achievement of energy balance. Two RCTs from a single center [13,14] reported tracheal aspiration. Patients tolerating a rate of at least 75% of the target calories within 60 hours were considered to have achieved energy balance in the study from Scotland [12] with only one patient converted to intravenous feeding from the NJ group. In contrast, in the study by Kumar *et al*. [13], the achievement of energy balance was defined by patients reaching a goal of 1,800 kcal within seven days from the start of feeding. Partial PN was necessary in only four and six patients in the NJ and NG groups, respectively. Patients achieving the goal nutrient requirement of 25 kcal/kg per day were considered to have achieved energy balance in the study by Singh *et al*. [14], and no additional PN was used. No heterogeneity (*P *= 0.64, 0.76, 0.51, 0.85, 1.00, respectively) was observed between the study results for all comparisons (Figures 2 to 6); therefore, a fixed effects model was used.

**Figure 7:**
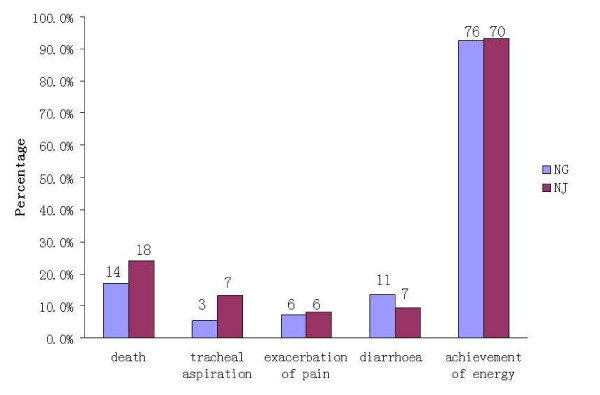
**The number and rates of death, tracheal aspiration, exacerbation of pain, diarrhea, and achievement of energy balance in the nasogastric and nasojejunal groups**. Numbers above the bars indicate the number of incidences in the patients in each group. No significant difference was found between the two groups.

The number of deaths, tracheal aspiration, exacerbation of pain, diarrhea, and achievement of energy balance was 14 (17.1%), 3 (5.5%), 6 (7.3%), 11 (13.4%) and 76 (92.7%) in the early NG group, respectively. In the NJ group, the numbers were 18 (24.0%), 7 (13.2%), 6 (8%), 7 (9.3%) and 70 (93.3%), respectively (Figure 7). The mortality rate is consistent with previous reports. No cases required withdrawal of the enteral feeding due to recurrent re-feeding pain. There were no significant differences in the incidence of mortality (RR = 0.69, 95% CI: 0.37 to 1.29, *P *= 0.25, Figure 2); tracheal aspiration (RR = 0.46, 95% CI: 0.14 to 1.53, *P *= 0.20, Figure 3); exacerbation of pain (RR = 0.94, 95% CI: 0.32 to 2.70, *P *= 0.90, Figure 4); diarrhea (RR = 1.43, 95% CI: 0.59 to 3.45, *P *= 0.43, Figure 5); or achievement of energy balance (RR = 1.00, 95% CI: 0.92 to 1.09, *P *= 0.97, Figure 6) between the NG and NJ feeding groups.

There were differences between patients in the studies from Scotland and India with respect to gender and etiology (*P *= 0.02, 0.02, respectively) (Table 6).

**Table 6:** Gender parameters of patients and underlying cause of pancreatitis in the two centers

Parameters	Scotland (number = 49)	India (number = 108)	*P*
Gender			0.02
Male	26	78	
Female	23	30	
Etiology			
Biliary	32	46	
Alcohol	12	30	0.02
Idiopathic	3	25	
Others	2	7	

**Figure 8:**
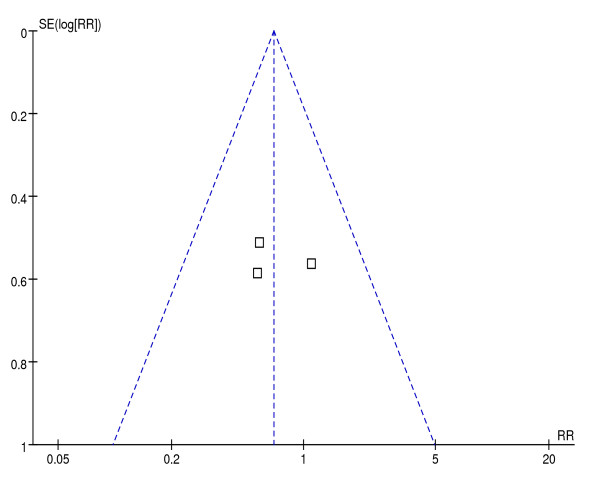
**Funnel plot for publication bias**.

Visual inspection of the funnel plot (Figure 8) did not indicate a publication bias.

## Discussion

Outcomes based on the analysis of the data from the three included RCTs were within the expected range. Eatock *et al*. [24] used early NG feeding in the nutritional management of SAP, followed by oral re-feeding [25] in patients with predicted SAP. It has been shown that NG feeding is feasible in up to 80% of cases [12]. Similarly, our meta-analysis showed that the safety and tolerance were not significantly different between the NG and NJ feeding groups, with no increase in mortality or nutrition-associated adverse events. As shown in Table 3, there was no significant difference between the NG and NJ feeding groups at admission with respect to MOF and IPN. Because primary or secondary infection of necrotized areas by enteral bacteria is considered a primary cause of mortality in patients with SAP, which is characterized by rapidly progressive MOF [26], we can conclude that the severity of the disease in the NG and NJ feeding groups was equivalent at admission, validating the comparison of NG and NJ feeding. APACHE II scores can provide better prediction of mortality in patients with SAP [27]. Similar to MOF and IPN, APACHE II scores in the NG and NJ feeding groups at admission were comparable in all of the included studies (Table 4 and 5, respectively), further validating our analysis.

NG or duodenal feeding has been believed to increase the chances of aspiration pneumonitis [16], but our results showed no significant difference between NG and NJ feeding with respect to tracheal aspiration, suggesting that NG feeding is as safe as NJ feeding. Also, Marik *et al*. [15] demonstrated no benefit from post-pyloric versus gastric tube feeding in a mixed group of critically ill patients with respect to tracheal aspiration. Delayed gastric emptying leads to impaired upper digestion and results in some degree of upper digestive intolerance [28]. Also, placement of small bowel feeding tubes using the blind nasoenteric approach is technically challenging and not as convenient or as easy as the placement of gastric feeding tubes. Misplacement of small bore feeding tubes into the lung with resultant pneumothorax is not a rare complication [15]. Although both fluoroscopy and endoscopy are highly effective for placement of small bowel feeding tubes, both techniques are expensive and inconvenient [15].

The effect of nutrition on pancreatic exocrine function is one of the most important issues concerning NG feeding in acute pancreatitis because 'pancreatic rest' is believed to promote healing, decrease pain, and reduce pancreatic secretions [29]. It has been shown that significantly higher secretions of trypsin (*P *<0.01) and lipase (*P *<0.05) occur in response to the elemental formula delivered into the duodenum compared to the jejunum (40 cm or more distal to the ligament of Treitz) in healthy subjects [17]. However, convincing evidence has shown that pancreatic exocrine function is significantly stronger in healthy subjects compared with patients with acute pancreatitis and suggests that the severity of acute pancreatitis is inversely related to duodenal secretion of pancreatic enzymes [30]. Therefore, a more likely alternative explanation for our findings that the safety and tolerance were not significantly different between the two nutrient feeding routes is that the pancreas becomes less responsive to NG stimulation during an attack of predicted SAP. Increased pancreatic secretion aggravates pancreatitis and leads to the exacerbation of pain. However, we found no significant difference between NG and NJ feeding with respect to the exacerbation of pain. Also, only two patients in the NG groups [12] in all of the included studies required non-opiate analgesia for pain and the others required no analgesia for re-feeding pain. Therefore, it is logical to speculate that the degree of re-feeding pain was not high according to the GRADE (Grading Assessment, Development and Evaluation) framework [31].

Hypermetabolism, with increased resting energy expenditure, has been demonstrated in patients with acute pancreatitis making nutritional supplements necessary along with other treatments. No difference in the achievement of energy balance in our analysis indicated that NG feeding was not inferior to NJ feeding.

Our study has a number of limitations. First, similar to the study by Petrov *et al*. [18], the number of subjects in our meta-analysis was small. Given the absence of robust power to confirm the results of their meta-analysis, Petrov *et al*. systematically reviewed NG feeding in patients with predicted SAP and demonstrated the necessity to support the NG approach by adequately powered randomized trials of NG versus NJ feeding. On the basis of our findings, following the recommendations of the study by Zhang *et al*. [32], we calculated that the number of subjects required to conduct an adequately powered non-inferiority trial was 864. Based on this calculation, our analysis was insufficient to detect any difference or to prove equivalence between the NG and NJ groups with respect to clinical outcomes. However, using a multi-center investigation would provide an adequate number of subjects. Also, given the large number of patients in the RCT by Singh *et al*. [14] compared with the other two RCTs [12,13], we believe that the addition of the third RCT did substantially increase the power and the precision of our meta-analysis. Second, blinding was not performed in any of the trials due to the nature of the interventions, which increased the bias. However, the assessed quality of the included RCTs was good. Third, because two of the included studies [13,14] originated from the same center in India, we analyzed the differences in gender and etiology between the centers in Scotland and India and unfortunately, found differences. However, we do not believe that this greatly decreased the power of the analysis to substantiate the conclusion that NG feeding is safe and well tolerated compared with NJ feeding. Although the underlying cause of the pancreatitis is important in determining the therapy, nutritional support is necessary in SAP regardless of etiology [33] and the emphasis has now shifted to early EN [34]. A considerable delay in commencing EN in each nutrient feeding route occurred in the RCT from India [14], resulting in a potential selection bias. Finally, our funnel plot should be interpreted with considerable caution given the small number of studies and patients. Larger studies are required to confirm our results, because plotting against precision (1/standard error) emphasizes differences between larger studies [35] and the capacity of funnel plots to detect bias is limited when meta-analyses are based on a limited number of small trials.

## Conclusions

In conclusion, although the evidence is not convincing, our meta-analysis demonstrated that NG feeding is safe and well tolerated compared with NJ feeding with respect to the mortality rate, tracheal aspiration, diarrhea, exacerbation of pain, and achievement of energy balance in patients with predicted SAP. EN by NG appears to provide an alternative to NJ feeding considering the similar outcomes and convenience. More high-quality, large-scale, RCTs are needed to validate the use of NG versus NJ feeding because our review is limited by the small total sample size and other limitations.

## Key messages

• NG feeding is safe and well-tolerated compared with NJ feeding.

• NG feeding appears to be an alternative to NJ feeding given the similar outcomes and convenience.

## Abbreviations

APACHE: Acute Physiology and Chronic Health Evaluation; EN: enteral nutrition; GRADE: Grading Assessment: Development and Evaluation; IPN: infected pancreatic necrosis; LOS: length of hospital stay; MOF: multiple organ failure; NG: nasogastric; NJ: nasojejunal; PN: parenteral nutrition; RCTs: randomized controlled trials; RR: risk ratio; CIs: confidence intervals; SAP: severe acute pancreatitis.

## Competing interests

The authors declare that they have no competing interests.

## Authors' contributions

All authors conceived the study and contributed to the study design. YSC collected data, performed the analyses, and drafted the paper. YMX performed the analyses and helped to extract data. JCL and QHF performed the literature review. All authors contributed to writing a draft and read and approved the final manuscript.
